# Socioeconomic disparities in first stroke incidence, quality of care, and survival: a nationwide registry-based cohort study of 44 million adults in England

**DOI:** 10.1016/S2468-2667(18)30030-6

**Published:** 2018-03-15

**Authors:** Benjamin D Bray, Lizz Paley, Alex Hoffman, Martin James, Patrick Gompertz, Charles D A Wolfe, Harry Hemingway, Anthony G Rudd, SSNAP Collaboration

**Affiliations:** a Farr Institute of Health Informatics Research, University College London, London, UK; b Sentinel Stroke National Audit Programme, Royal College of Physicians, London, UK; c Royal Devon and Exeter NHS Foundation Trust, Exeter, UK; d National Cardiovascular Intelligence Network, Public Health England, London, UK; e School of Population Health & Environmental Sciences, King's College London, London, UK

## Abstract

**Background:**

We aimed to estimate socioeconomic disparities in the incidence of hospitalisation for first-ever stroke, quality of care, and post-stroke survival for the adult population of England.

**Methods:**

In this cohort study, we obtained data collected by a nationwide register on patients aged 18 years or older hospitalised for first-ever acute ischaemic stroke or primary intracerebral haemorrhage in England from July 1, 2013, to March 31, 2016. We classified socioeconomic status at the level of Lower Super Output Areas using the Index of Multiple Deprivation, a neighbourhood measure of deprivation. Multivariable models were fitted to estimate the incidence of hospitalisation for first stroke (negative binomial), quality of care using 12 quality metrics (multilevel logistic), and all-cause 1 year case fatality (Cox proportional hazards).

**Findings:**

Of the 43·8 million adults in England, 145 324 were admitted to hospital with their first-ever stroke: 126 640 (87%) with ischaemic stroke, 17 233 (12%) with intracerebral haemorrhage, and 1451 (1%) with undetermined stroke type. We observed a socioeconomic gradient in the incidence of hospitalisation for ischaemic stroke (adjusted incidence rate ratio 2·0, 95% CI 1·7–2·3 for the most *vs* least deprived deciles) and intracerebral haemorrhage (1·6, 1·3–1·9). Patients from the lowest socioeconomic groups had first stroke a median of 7 years earlier than those from the highest (p<0·0001), and had a higher prevalence of pre-stroke disability and diabetes. Patients from lower socioeconomic groups were less likely to receive five of 12 care processes but were more likely to receive early supported discharge (adjusted odds ratio 1·14, 95% CI 1·07–1·22). Low socioeconomic status was associated with a 26% higher adjusted risk of 1-year mortality (adjusted hazard ratio 1·26, 95% CI 1·20–1·33, for highest *vs* lowest deprivation decile), but this gradient was largely attenuated after adjustment for the presence of pre-stroke diabetes, hypertension, and atrial fibrillation (1·11, 1·05–1·17).

**Interpretation:**

Wide socioeconomic disparities exist in the burden of ischaemic stroke and intracerebral haemorrhage in England, most notably in stroke hospitalisation risk and case fatality and, to a lesser extent, in the quality of health care. Reducing these disparities requires interventions to improve the quality of acute stroke care and address disparities in cardiovascular risk factors present before stroke.

**Funding:**

NHS England and the Welsh Government.

## Introduction

Ischaemic stroke and intracerebral haemorrhage are leading causes of non-communicable disease, together affecting an estimated 17 million people and contributing to 5·9 million deaths globally each year.^1^ It has long been recognised that the burden of stroke is not evenly distributed in society, and that major socioeconomic disparities exist in ischaemic stroke risk both between^1^ and within^2^ countries. Previous systematic reviews^2^, ^3^ have identified gaps in knowledge of the links between socioeconomic status—ie, an individual's or group's position in society, encompassing a range of factors such as income, education, employment, and social status—and stroke. These uncertainties include whether, and to what extent, socioeconomic status is associated with risk of intracerebral haemorrhage; whether or not disparities exist in the provision of good-quality stroke care; and what the causal mechanisms are for the association between low socioeconomic status and increased risk of ischaemic stroke.^2^ Addressing these gaps would not only help to better quantify and characterise the links between socioeconomic status and stroke, but also help to guide efforts to reduce the burden of stroke through health service development and primary prevention.

Real-world electronic health record and population datasets are providing new ways to address these types of epidemiological questions at a scale not possible with traditional study designs or data sources.^4^ Using detailed clinical data collected and linked to other datasets through a nationwide stroke register, we aimed to estimate for the whole adult population of England the association between socioeconomic status and the risk of hospitalisation for first-ever ischaemic stroke and intracerebral haemorrhage, identify disparities across a comprehensive range of acute stroke-care quality metrics, and estimate disparities in survival up to 1 year after stroke.

**Research in context**

**Evidence before this study**

We searched PubMed with the terms [“Stroke” OR “Ischaemic stroke” OR “Intracerebral haemorrhage”] AND [“Disparities” OR “Inequalities” OR “Socioeconomic” OR “Socioeconomic status”]. We found three high-quality systematic reviews and a large number of primary research studies. The association between low socioeconomic status and an increased risk of ischaemic stroke is well established and has been shown in many previous studies. However, there is conflicting evidence on whether socioeconomic status is linked to the risk of intracerebral haemorrhage and whether socioeconomic status is associated with disparities in care and outcomes after stroke. There is also uncertainty about the causal mechanisms between socioeconomic status and stroke risk and hence what can be done to reduce disparities in risk. We found several previous registry-based studies but no studies that sought to describe for a whole country the associations between socioeconomic status and a wide spectrum of stroke-related outcomes encompassing incidence, quality of care, and outcomes.

**Added value of this study**

We found strong evidence for a socioeconomic gradient in the risk of both ischaemic stroke and intracerebral haemorrhage, and found that the gradient was steeper for ischaemic stroke than for intracerebral haemorrhage. Patients from low socioeconomic groups had stroke a median of 7 years earlier than those with high socioeconomic status but, despite being younger, had a higher prevalence of pre-stroke disability. We found evidence of a strong gradient in the risk of ischaemic stroke with pre-existing diabetes and to a lesser extent with pre-existing hypertension and atrial fibrillation. Low socioeconomic status was associated with poorer care for five of 12 care-quality metrics and better care for one metric when compared with patients from the least deprived areas. Low socioeconomic status was associated with higher 1-year case fatality, but this association was largely attenuated after adjusting for pre-stroke cardiovascular risk factors.

**Implications of all the available evidence**

This study provides evidence that socioeconomic status is associated with the risk of intracerebral haemorrhage in addition to ischaemic stroke, that diabetes might be a particularly important mediator of higher stroke risk in populations of low socioeconomic status, and that pre-stroke vascular risk factors might explain apparent disparities in post-stroke survival (up to the first year after stroke). Policy makers and care providers should address disparities both in the quality of health care and in health determinants present earlier in life before stroke. These findings have global implications in efforts to tackle one of the leading causes of non-communicable disease.

## Methods

### Stroke incidence, care, and survival data

We obtained data on patients with stroke from the Sentinel Stroke National Audit Programme (SSNAP), the national stroke register of England, Wales, and Northern Ireland. SSNAP includes patients admitted to hospital with acute ischaemic stroke or primary intracerebral haemorrhage. All acute admitting hospitals participate and data are submitted by clinical teams via a web-based portal with real-time data validation. Overall case ascertainment in SSNAP for hospitalised stroke is estimated to be 95%, comparing against hospital discharge coding and data collected locally by hospitals about stroke admission.

Quality of care was measured within SSNAP using 12 care-quality metrics chosen specifically from key indicators reported by SSNAP: thrombolysis with alteplase (if ischaemic stroke and presenting within 4·5 h of stroke onset); door-to-needle time of less than 60 min for thrombolysis; brain scan within 1 h of hospital arrival; screening for dysphagia within 4 h of hospital arrival; admission to a stroke unit or intensive care unit within 4 h of hospital arrival; physiotherapy and occupational and speech therapy assessment within 72 h of hospital arrival; assessment by a stroke specialist physician and stroke nurse within 24 h of hospital arrival; treatment with an anticoagulant by discharge or plan to start within 1 month of discharge (if ischaemic stroke and diagnosed with atrial fibrillation); and discharge to an early supported discharge team. For patients having an acute stroke while already a hospital inpatient, the time of stroke onset was used in place of time of arrival to calculate these quality metrics.

Because this study aimed to describe the incidence of hospitalisation for first stroke, we excluded patients with a previous diagnosis of stroke or transient ischaemic attack. The study cohort included patients in England aged 18 years or older admitted to hospital with first acute ischaemic stroke or first primary intracerebral haemorrhage between July 1, 2013, and March 31, 2016. We used a pseudonymised data extract with no patient identifiers included.

Mortality data were obtained by information provided by clinical teams and via patient-level record linkage to the national statutory register of death certification records (Office of National Statistics).

### Population data and neighbourhood economic status

Age-stratified (in 5-year bands) and sex-stratified mid-year population estimates were obtained from the Office of National Statistics^5^ to define the size and population structure of the denominator population. We did this at the level of Lower Super Output Areas (LSOAs; England is divided into 32 844 LSOAs, each comprising approximately 1500 individuals). We defined neighbourhood socioeconomic status using the Index of Multiple Deprivation (IMD), a multicomponent measure of neighbourhood deprivation.^6^ Each LSOA is ranked according to IMD score, with 1 being the most deprived neighbourhood in England. We aggregated the age and population structure of the LSOAs according to decile of IMD, and used these deciles for the statistical analysis and model fitting. We obtained data on included patients' socioeconomic status by linking their postcode of residence to the appropriate LSOA, and classified patients by the IMD decile of their neighbourhood.

SSNAP has permission from the National Health Service (NHS) Health Research Authority under section 251 of the Health and Social Care Act 2006 to collect patient data without prospective consent. Patients can opt out of data linkage with the national death register. Additional ethical permission was not sought.

### Statistical analysis

Incidence rate ratios were estimated separately for ischaemic stroke and primary intracerebral haemorrhage by fitting negative binomial regression models, adjusting for age and sex. We fitted further models to estimate incidence rate ratios for the combination of ischaemic stroke plus pre-stroke vascular comorbidity (hypertension, diabetes, and atrial fibrillation) to explore socioeconomic differences in ischaemic stroke risk factors.

We estimated quality of care by fitting multilevel logistic regression models with random intercepts to take account of clustering at the hospital level. Models were adjusted for age, sex, stroke type, onset in hospital, time from stroke onset to admission, independence before stroke (modified Rankin Scale score of 0–1),^7^ and stroke severity. Stroke severity was measured using the admission National Institutes of Health Stroke Scale (NIHSS) for patients with complete NIHSS data and level of consciousness on admission for the remainder.

We estimated survival up to 1 year after admission (or after stroke onset if inpatient at time of stroke) by fitting Cox proportional hazards models, using the robust sandwich estimator to take account of clustering at the hospital level. The proportional hazards assumption was checked for all covariates using log–log plots. We fitted two sets of models: first, models adjusting only for baseline demographic variables (age, sex, and stroke type), and then models also adjusting for pre-stroke comorbidities (hypertension, diabetes, and atrial fibrillation).

We did all analyses using Stata 14 MP and Python 3.6 (Numpy 1.12, Matplotlib 2.0, Pandas 0.20).

### Role of the funding source

The study funders had no role in the design of the study, data collection, analysis, data interpretation, or writing of the report. BDB and LP had access to the raw data. The corresponding author had full access to all of the data and the final responsibility to submit for publication.

## Results

Over the 33 months of the study, among the 43·8 million adults in England, 145 324 were admitted to hospital with their first-ever stroke. Of these, 126 640 (87%) were admitted with first ischaemic stroke, 17 233 (12%) with intracerebral haemorrhage, and 1451 (1%) with undetermined stroke type (table 1). The crude incidence rate was 105 (95% CI 104–106) per 100 000 person-years for hospitalisation for first ischaemic stroke and 14 (14–15) per 100 000 person-years for first intracerebral haemorrhage.

**Table 1:** Characteristics of the cohort

		**Data**
Adults aged ≥18 years in England		43 749 578
Total person-years in cohort		120 311 340
Admission for first-ever stroke		145 324
Stroke type		
	Ischaemic stroke	126 640 (87%)
	Intracerebral haemorrhage	17 233 (12%)
	Undetermined	1451 (1%)
Sex		
	Female	72 412 (50%)
	Male	72 912 (50%)
Age, years		77 (66–85)
Pre-existing comorbidity		
	Hypertension	75 130 (52%)
	Atrial fibrillation	26 459 (18%)
	Diabetes	27 119 (19%)
NIHSS fully complete on admission		118 066 (81%)
NIHSS		4 (2–10)
Level of consciousness on admission		
	Alert	121 696 (84%)
	Responds to voice	13 847 (10%)
	Responds to pain	5722 (4%)
	Unconscious	4059 (3%)
Independent before stroke (mRS 0–1)		109 252 (75%)
Time from onset to arrival		
	0 h to <3 h	47 984 (33%)
	3 h to <6 h	14 066 (10%)
	≥6 h	27 510 (19%)
	Unknown (eg, wake up stroke)	47 652 (33%)
	Onset in hospital	8112 (6%)
All-cause 30-day mortality		20 157 (14%)
All-cause 1-year mortality if follow-up >365 days		28 434/107 891 (26%)

Data are n, n (%), n/N (%), or median (IQR). Percentages are calculated on the total number of admissions for first-ever stroke, unless stated otherwise. NIHSS=National Institutes of Health Stroke scale. mRS=modified Rankin scale.

Patient characteristics differed across the socioeconomic gradient. Patients from the most deprived areas had a median age of stroke onset 7 years younger than those in the least deprived areas (table 2; appendix p 3). Despite being younger, patients from the most deprived areas were less likely to be independent before stroke and had a higher crude prevalence of pre-stroke diabetes than did patients from t he least deprived areas (table 2). By contrast, patients from the most deprived areas had a lower crude prevalence of pre-stroke atrial fibrillation than did patients from the least deprived areas (table 2).

**Table 2:** Characteristics of patients with stroke by decile of Index of Multiple Deprivation

		**10 (least deprived)**	**9**	**8**	**7**	**6**	**5**	**4**	**3**	**2**	**1 (most deprived)**	**p value**
Patients with stroke		13 434	14 256	14 611	15 280	15 408	15 152	14 564	14 170	14 076	14 373	..
Stroke type												<0·0001*
	Ischaemic	11 542 (86%)	12 315 (86%)	12 699 (87%)	13 262 (87%)	13 450 (87%)	13 256 (87%)	12 734 (87%)	12 364 (87%)	12 347 (88%)	12 671 (88%)	..
	Haemorrhagic	1755 (13%)	1815 (13%)	1792 (12%)	1874 (12%)	1804 (12%)	1765 (12%)	1694 (12%)	1628 (11%)	1547 (11%)	1559 (11%)	..
	Undetermined	137 (1%)	126 (1%)	120 (1%)	144 (1%)	154 (1%)	131 (1%)	136 (1%)	178 (1%)	182 (1%)	143 (1%)	..
Sex												<0·0001*
	Female	6725 (50%)	7079 (50%)	7374 (50%)	7563 (49%)	7836 (51%)	7542 (50%)	7297 (50%)	7143 (50%)	6971 (50%)	6882 (48%)	..
	Male	6709 (50%)	7177 (50%)	7237 (50%)	7717 (51%)	7572 (49%)	7610 (50%)	7267 (50%)	7027 (50%)	7105 (50%)	7491 (52%)	..
Age, years		79 (69–86)	78 (68–86)	78 (68–85)	78 (68–85)	78 (67–85)	77 (66–85)	76 (65–84)	75 (63–84)	73 (61–83)	72 (60–81)	<0·0001†
Hypertension		6979 (52%)	7340 (52%)	7542 (52%)	7888 (52%)	7996 (52%)	7809 (52%)	7591 (52%)	7240 (51%)	7355 (52%)	7390 (51%)	0·70*
Atrial fibrillation		2725 (20%)	2848 (20%)	2927 (20%)	3079 (20%)	3055 (20%)	2724 (18%)	2537 (17%)	2311 (16%)	2155 (15%)	2053 (14%)	<0·0001*
Diabetes		1924 (14%)	2266 (16%)	2399 (16%)	2630 (17%)	2793 (18%)	2861 (19%)	2874 (20%)	2932 (21%)	3130 (22%)	3310 (23%)	<0·0001*
Independent before stroke		10 397 (77%)	10 959 (77%)	11 042 (76%)	11 539 (76%)	11 510 (75%)	11 343 (75%)	10915 (75%)	10 397 (73%)	10 485 (74%)	10 665 (74%)	<0·0001*
NIHSS on admission (n=118 066)		4 (2–10)	4 (2–10)	4 (2–10)	4 (2–10)	4 (2–10)	4 (2–10)	4 (2–10)	5 (2–10)	4 (2–10)	4 (2–10)	<0·0001†
Level of consciousness on admission												0·019*
	Alert	11 262 (84%)	11 989 (84%)	12 174 (83%)	12 749 (83%)	12 813 (83%)	12 624 (83%)	12 196 (84%)	11 850 (84%)	11 861 (84%)	12 178 (85%)	..
	Responds to voice	1269 (9%)	1330 (9%)	1444 (10%)	1480 (10%)	1542 (10%)	1470 (10%)	1387 (10%)	1381 (10%)	1291 (9%)	1253 (9%)	..
	Responds to pain	551 (4%)	530 (4%)	607 (4%)	616 (4%)	596 (4%)	579 (4%)	594 (4%)	552 (4%)	547 (4%)	550 (4%)	..
	Unconscious	352 (3%)	407 (3%)	386 (3%)	435 (3%)	457 (3%)	479 (3%)	387 (3%)	387 (3%)	377 (3%)	392 (3%)	..
Onset to arrival time												<0·0001*
	0 h to <3 h	4765 (35%)	4993 (35%)	5168 (35%)	5109 (33%)	5105 (33%)	4979 (33%)	4755 (33%)	4570 (32%)	4320 (31%)	4220 (29%)	..
	3 h to <6 h	1407 (10%)	1433 (10%)	1403 (10%)	1495 (10%)	1491 (10%)	1478 (10%)	1394 (10%)	1307 (9%)	1415 (10%)	1243 (9%)	..
	≥6 h	2566 (19%)	2689 (19%)	2597 (18%)	2842 (19%)	2808 (18%)	2807 (19%)	2724 (19%)	2712 (19%)	2802 (20%)	2963 (21%)	..
	Unknown (eg, wake up stroke)	3951 (29%)	4354 (31%)	4659 (32%)	4954 (32%)	5154 (33%)	5023 (33%)	4872 (33%)	4830 (34%)	4767 (34%)	5088 (35%)	..
	Onset in hospital	745 (6%)	787 (6%)	784 (5%)	880 (6%)	850 (6%)	865 (6%)	819 (6%)	751 (5%)	772 (5%)	859 (6%)	..
All-cause 30-day mortality		1902 (14%)	1953 (14%)	2120 (15%)	2231 (15%)	2246 (15%)	2182 (14%)	1997 (14%)	1921 (14%)	1760 (13%)	1845 (13%)	<0·0001*
All-cause 1-year mortality if follow-up >365 days (n=107 891)		2650/9895 (27%)	2819/10 545 (27%)	2852/10 818 (26%)	3163/11 344 (28%)	3127/11 466 (27%)	3030/11 168 (27%)	2837/10 757 (26%)	2738/10 541 (26%)	2597/10 622 (24%)	2621/10 735 (24%)	<0·0001

Data are n, n (%), or median (IQR). Data are for all 145 324 patients admitted for first ever stroke unless stated otherwise. NIHSS=National Institutes of Health Stroke scale.

* By χ^2^ test.

† By Kruskal Wallis test.

**Figure 1:**
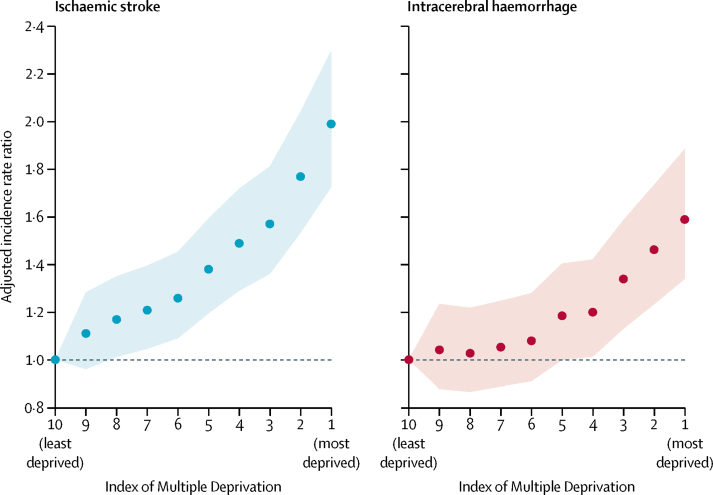
Age-adjusted and sex-adjusted incidence rate ratio for ischaemic and intracerebral haemorrhage stroke Shaded regions indicates 95% CIs.

**Figure 2:**
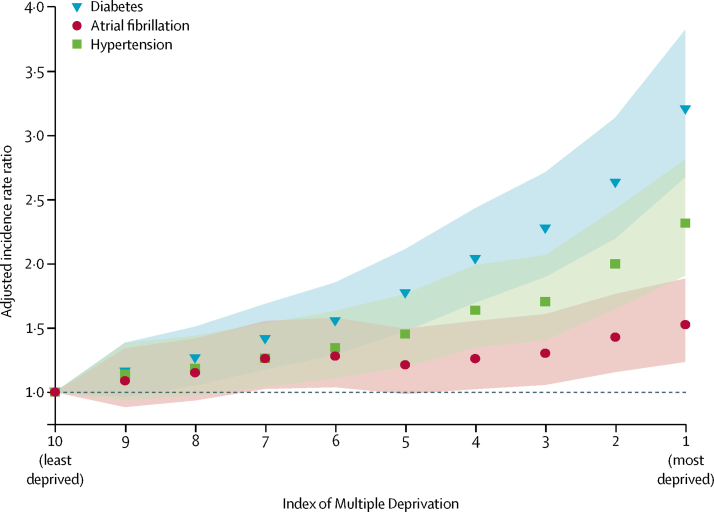
Age-adjusted and sex-adjusted incidence rate ratio of combination of ischaemic stroke with pre-existing comorbidities Shaded regions indicate 95% CIs.

Lower socioeconomic status was associated with a higher age-adjusted and sex-adjusted incidence of both first ischaemic stroke and first intracerebral haemorrhage (figure 1; appendix). Compared with people in the least deprived areas, those living in the most deprived areas had twice the age-adjusted and sex-adjusted incidence rate (aIRR 2·0, 95% CI 1·7–2·3) of ischaemic stroke and 1·6 (95% CI 1·3–1·9) times the aIRR of intracerebral haemorrhage (figure 1); a map of aIRRs of ischaemic stroke by LSOA is provided in the appendix (p 5). We observed similar gradients in the proportion of patients with first stroke and pre-existing cardiovascular comorbidities (figure 2; appendix p 4). People in the most deprived areas had 3·2 (2·7–3·8) times the aIRR of ischaemic stroke plus pre-existing diabetes, 2·3 (1·9–2·8) times the aIRR of ischaemic stroke plus pre-existing hypertension, and 1·5 (1·2–1·9) times the aIRR of ischaemic stroke plus pre-existing atrial fibrillation (figure 2).

**Figure 3:**
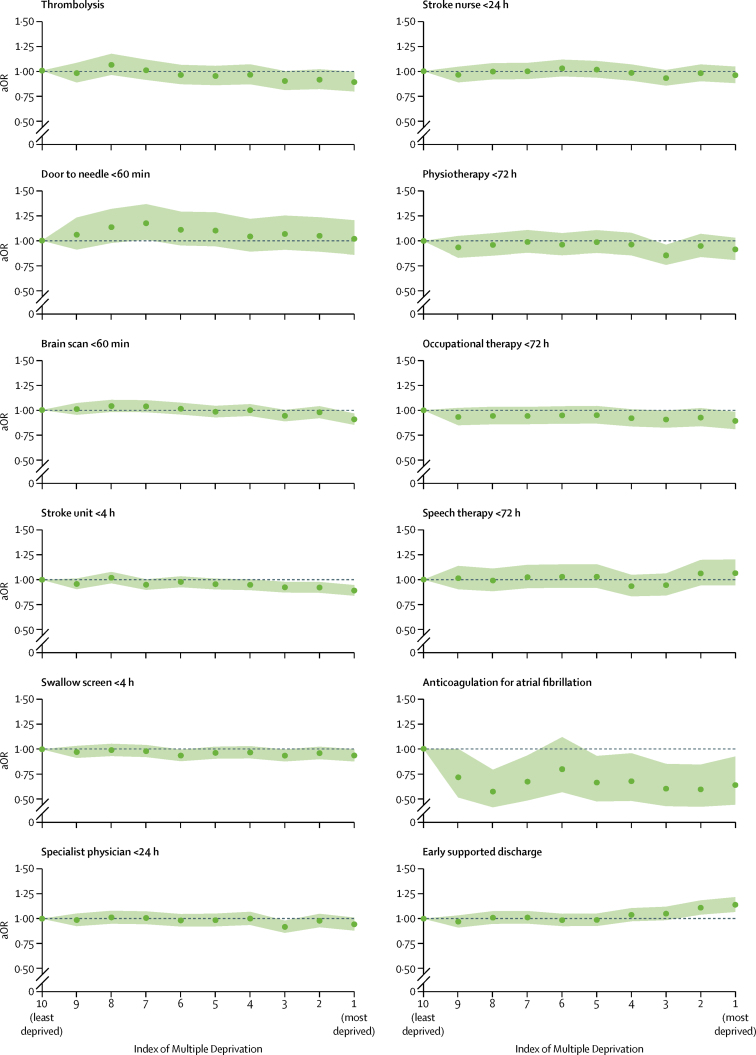
Adjusted odds of 12 measures of stroke-care quality Shaded regions indicate 95% CIs. aOR=adjusted odds ratio.

Overall, quality of care was fairly equal, but some aspects of care quality varied with socioeconomic status (figure 3). We found no evidence of disparities in door-to-needle times for thrombolysis, swallow screen within 4 h, specialist physician and stroke nurse within 24 h, or physiotherapy and speech therapy assessments within 72 h. We found moderately strong evidence that patients from the most deprived areas were less likely to receive thrombolysis (adjusted odds ratio [aOR] 0·89, 95% CI 0·80–0·99 using the least deprived decile as the reference category), have a brain scan within 1 h of arrival (0·91, 0·86–0·97), receive an occupational therapy assessment within 72 h (0·89, 0·81–0·98), or be admitted to a stroke unit within 4 h (0·89, 0·84–0·95). Compared with patients from the least deprived areas, all groups were less likely to receive anticoagulation for atrial fibrillation (aOR 0·64, 0·44–0·92 for the most deprived decile *vs* the least deprived decile; figure 3). By contrast with the disparities observed for other aspects of care, patients living in the most deprived areas had a greater adjusted odds of being discharged to an early supported discharge team (1·14, 1·07–1·22; figure 3).

The crude 1-year case fatality rate for the whole cohort was 26%. Adjusting for differences in age, sex, and stroke type, we observed a gradient in mortality risk after stroke, with patients in the most deprived areas having a 26% higher risk of death in the first year after stroke compared with patients from the least deprived areas (table 3; appendix p 6). This gradient was largely but not completely attenuated after additionally adjusting for pre-stroke hypertension, atrial fibrillation, and diabetes, with the point estimates for the hazard ratios shrinking towards 1. In the latter model, there was strong evidence from the 95% CIs that only patients from the most deprived decile had a higher risk of death compared with the patients from the least deprived decile (table 3).

**Table 3:** Adjusted hazard ratios (95% CI) for 1-year all-cause mortality

	**10 (least deprived)**	**9**	**8**	**7**	**6**	**5**	**4**	**3**	**2**	**1 (most deprived)**
Adjusted for age, sex, and stroke type	1 (ref)	1·02 (0·98–1·07)	1·03 (0·98–1·09)	1·09 (1·03–1·14)	1·10 (1·05–1·16)	1·13 (1·08–1·18)	1·12 (1·07–1·17)	1·17 (1·12–1·22)	1·17 (1·11–1·22)	1·26 (1·20–1·33)
Adjusted for age, sex, stroke type, and pre-stroke comorbidity	1 (ref)	1·00 (0·96–1·05)	0·99 (0·94–1·05)	1·04 (0·99–1·09)	1·03 (0·97–1·09)	1·05 (1·00–1·10)	1·04 (0·98–1·10)	1·05 (1·00–1·11)	1·04 (0·98–1·10)	1·11 (1·05–1·17)

## Discussion

This study drew on detailed registry data to estimate and map the associations between socioeconomic status and stroke incidence, quality of care, and post-stroke survival for the whole adult population of England. We found strong evidence that people living in more deprived areas have a higher risk of first-ever ischaemic stroke and intracerebral haemorrhage for which they are hospitalised and that they experience stroke earlier in life than do those in less deprived areas. Having stroke at a younger age has major implications for society and individuals, affecting individuals' ability to work,^8^ earn income, and live a life free from long-term disability. The association was most marked for the combination of ischaemic stroke and prior history of diabetes, suggesting that diabetes is an important mediator of higher ischaemic stroke risk in populations with lower socioeconomic status. Overall the quality of in-patient acute stroke care provided by the NHS in England was fairly equitable, but we found evidence of disparities in some aspects of care, including prescription of anticoagulation for atrial fibrillation and timely admission to a specialist stroke unit, one of the principal indicators of improved disability outcomes after stroke. Patients from the most deprived areas had lower 1-year survival compared with patients of similar age and sex from less deprived areas. These differences were largely but not completely attenuated after adjusting for three pre-stroke cardiovascular comorbidities, implying that apparent disparities in survival up to the first year after stroke can mainly be explained by differences in vascular risk identifiable prior to the index stroke. Reducing socioeconomic disparities would have a major effect on the high burden of stroke on individuals and society, and efforts to reduce disparities in stroke need to address not only access to good quality health care, but also the determinants of health and vascular risk earlier in life.

This study is consistent with a large number of studies finding that both neighbourhood-level^9^, ^10^ and person-level^11^ measures of low socioeconomic status are associated with a higher risk of ischaemic stroke. These associations extend across the life course from the earliest years, and childhood socioeconomic status and education^12^ are associated with the lifetime risk of stroke. Although previous studies^13^, ^14^, ^15^ have not consistently shown an association with the risk of intracerebral haemorrhage, we found strong evidence of a gradient in intracerebral haemorrhage risk, albeit not as marked as that for ischaemic stroke. Previous studies^13^, ^14^, ^15^ have included only small numbers of patients with intracerebral haemorrhage and so might have been underpowered to detect this association. We are not aware of previous studies that have specifically estimated the incidence of ischaemic stroke plus previous risk factors, but increased prevalence of diabetes in patients with stroke of lower socioeconomic status has been previously reported in a population-based study from Ontario, Canada,^16^ a pooled analysis of three population-based studies in Australia and New Zealand,^17^ and a nationwide registry-based study from Denmark.^11^ In addition to differences in diabetes prevalence, it is also possible that disparities in the quality of diabetes care (eg, adequate blood pressure control) might contribute to the gradient in stroke risk. We also found evidence of a steep gradient in the incidence rate of ischaemic stroke plus previous history of hypertension and a more modest gradient in ischaemic stroke plus atrial fibrillation. The role of these risk factors in stroke aetiology is well established,^18^, ^19^ but our findings imply that interventions to improve the prevention and clinical management of diabetes might have a particularly large effect on reducing disparities in stroke risk.

Previous studies^2^, ^16^, ^20^, ^21^, ^22^ have found conflicting evidence of socioeconomic disparities in care. Little agreement between studies is not surprising because disparities are likely to be specific to individual health systems, limiting the extent to which findings can be generalised between settings. However, previous studies have shown, as with this study, that even high-income countries with universal health-care systems can have disparities in acute stroke care. A nationwide study^20^ in Denmark of 14 545 patients found that lower socioeconomic status was associated with lower achievement of seven care-quality processes. Similarly, patients in Sweden with lower levels of educational attainment were found to have lower odds of receiving reperfusion therapy after stroke^21^ and being prescribed oral anticoagulation^22^—a similar pattern to that found in our study using a different measure of socioeconomic status. The only previous nationwide study^23^ in England of socioeconomic disparities in stroke care used administrative data to describe variation in one stroke quality marker and found evidence that the most deprived patients were less likely to receive a brain scan on the same day of admission. Although five of the 12 quality measures were less likely to be achieved in patients of lower socioeconomic status, we identified one quality marker (early supported discharge) that was achieved more consistently in patients of lower socioeconomic status. It is not clear why this was the case, but possible explanations include differences between rural and urban provision of early supported discharge or personal factors (such as having a family member at home able to provide support) correlated with socioeconomic status. The reasons for the observed disparities in care quality are unclear, but it seems unlikely that financial barriers to accessing health care are a major factor, given that the NHS in England provides health care free at the point of use and is funded through general taxation. Identification of the mechanisms for these disparities would help to inform quality improvement interventions to reduce these disparities in care.

Our findings of lower survival in the first year after stroke with greater levels of socioeconomic deprivation are consistent with many^20^, ^24^, ^25^, ^26^ but not all^17^ previous studies. We also found that adjusting for pre-stroke vascular risk factors largely accounted for the observed socioeconomic gradient in survival. This provides evidence that socioeconomic differences in post-stroke survival in the first year after stroke are driven to a large extent by risk factors present before stroke. Simply improving the care that patients with acute stroke receive is therefore necessary but not sufficient to redress socioeconomic disparities in survival and, as with stroke risk, will require population-level and individual-level public health and behaviour change interventions to prevent and modify cardiovascular risk factors earlier in life.

This study has several strengths. To our knowledge, it is, by a large margin, the largest ever study of the associations between socioeconomic status and stroke-related outcomes, providing greater statistical power to estimate these associations and helping to address previous uncertainties in the epidemiology of stroke. It has also made use of detailed registry data to describe multiple elements of stroke care quality that would not be measurable in other big data sources such as administrative data. Using a whole country as a sampling frame has the advantage of reducing the risk of ascertainment bias and makes the findings potentially more relevant to policy makers, allowing for whole country estimates to be generated and mapped (eg, as done in the appendix maps). However, the study also has some limitations. First, this study used an area-based measure of socioeconomic status rather than person-level measures of socioeconomic status (such as income or level of educational attainment). This means that caution needs to be used in attributing group-level estimates to individuals (the ecological fallacy). Although this study made use of clinically validated registry data rather than administrative data, the dataset did not contain information about some important stroke risk factors,^18^, ^19^ such as smoking status, body-mass index, or physical activity. Outcome data were limited to mortality, but we recognise that other outcomes (eg, disability) are very important to stroke survivors and would have included these had data been available. Similarly, the quality metrics largely related to acute stroke care and suitable metrics about the quality of community-based or follow-up services were not available. We also did not have sufficiently detailed (age-stratified and sex-stratified) data about the ethnicity profile of LSOAs to take account of the effect of ethnicity on stroke risk (appendix). Similarly, although the estimated case ascertainment of SSNAP is high (approximately 95%), using data collected as part of routine care means that case ascertainment is less complete than would be achieved by an ideal population-based register with multiple overlapping sources of notification and a dedicated system of data collection.^27^ The estimates from this study do not include patients not admitted to hospital (a previous study^28^ from the UK reported that 12% of patients with stroke were not admitted to hospital) or patients with transient ischaemic attack: the sum effect is therefore that the incidence rates estimated in this study are likely to underestimate the total population burden of stroke and are lower than incidence studies that include patients with acute stroke who are not admitted to hospital.^29^

In conclusion, we found evidence of a wide range of socioeconomic disparities in stroke incidence, quality of care, and survival. Tackling these disparities would contribute substantially to reducing the burden of stroke on individuals and society, and should aim to address both the quality of acute stroke care and, to a larger extent, the determinants of cardiovascular risk earlier in life.
