# Supplementary material for: Socioeconomic disparities in first stroke incidence, quality of care, and survival: a nationwide registry-based cohort study of 44 million adults in England
Source: Lancet Public Health. 2018 Mar 15;3(4):e185–93. doi: 10.1016/S2468-2667(18)30030-6 (PMC5887080; doi:10.1016/S2468-2667(18)30030-6)
Supplement: Supplementary appendix [file mmc1.pdf]

THE LANCET
Public Health

Supplementary appendix

This appendix formed part of the original submission and has been peer reviewed. We post it as supplied by the authors.

Supplement to: Bray BD, Paley L, Hoffman A, et al, on behalf of the SSNAP Collaboration. Socioeconomic disparities in first stroke incidence, quality of care, and survival: a nationwide registry-based cohort study of 44 million adults in England. *Lancet Public Health* 2018; published online March 14. http://dx.doi.org/10.1016/S2468-2667(18)30030-6.

Appendix

Methods of Data Collection in SSNAP

SSNAP collects data through a secure web based portal and by upload of data from Electronic Healthcare Record systems. Data are provided by clinical teams and each hospital and community based stroke team has a designated lead (usually a clinical lead) responsible for the quality of the data. Data entry is supported by real time automated data validation checks (e.g. to not allow impossible or inconsistent data and to prompt users to check implausible or unlikely data), and regular feedback to centres.

SSNAP collects and reports a range of data including patient characteristics, stroke phenotype, quality of care metrics and patient outcomes. The current dataset is available at: www.strokeaudit.org/SupportFiles/Documents/Clinical-Datasets-and-Help-Notes/SSNAP-Core-Dataset-4-0-0.aspx and help notes and data definitions are available at https://www.strokeaudit.org/SupportFiles/Documents/Clinical-Datasets-and-Help-Notes/SSNAP-Helpnotes-for-core-dataset-4-0-0.aspx

SSNAP provides detailed guidance on definitions in order to ensure consistency of data entry and recording. The diagnosis of co morbidities (In this study: AF, diabetes, hypertension and prior stroke or transient ischaemic attack) prior to admission is pragmatic and based on the clinical data captured in the hospital patient records by the admitting clinical team. In most instances, information about co morbidities is derived from patient's electronic primary care or hospital health records. In this study, we did not include new diagnoses of diabetes, AF or hypertension made during the course of the hospital admission for acute stroke. Previous stroke was as classified by the admitting clinical team, and would typically be based on information in the patient's electronic primary care or hospital health records.

Case ascertainment

We looked for a possible association between socioeconomic status (SES) and case ascertainment in SSNAP, as a key assumption of the methodology for the incidence rates was that case ascertainment did not vary according to SES. Case ascertainment in SSNAP is measured by comparing against hospital discharge coding and information collected locally by hospitals about stroke admissions. Between 2013 and 2016 the estimated case ascertainment for SSNAP increased from 85% to over 90% .

It is not possible to measure case ascertainment at the level of individual LSOAs and so we measured case ascertainment at the level of Clinical Commissioning Groups (CCGs). CCGs are an organisational unit of the NHS and cover a defined population (co-terminus with the LSOAs that make up each CCG). We calculated the mean and standard deviation Index of Multiple Deprivation (higher is more deprived) for each CCG and tested the null hypothesis of no association between mean IMD and case ascertainment. We found no statistical evidence of an association (p = 0.16 using the Kruskal Wallis test). We also examined the distribution of IMD by case ascertainment band [Figure A1] using swarmplots to provide a visual check: again we found no evidence (on visual inspection) of an association

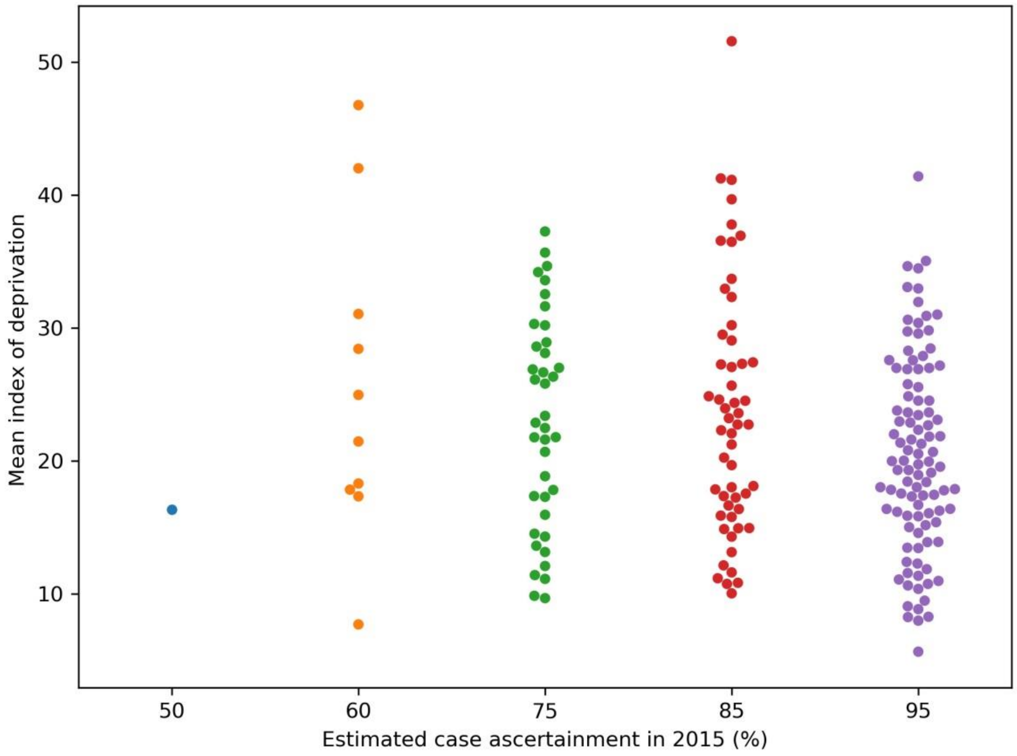

Figure A1. Distribution of mean index of multiple deprivation score by estimated case ascertainment. Data have been reported at the level of Clinical Commissioning Groups. Case ascertainment has been calculated in bands, and plotted as the mid point of the band, with the exception of the single CCG with an estimated case ascertainment of less than 50%)

Case ascertainment band in SSNAP (2015)	Mean (SD) Index of Multiple Deprivation
<50	17 (NA)
50-69	26 (12
70-79	23 (8)
80-89	24 (9)
90+	20 (7)

Table A1. Mean index of multiple deprivation by levels of case ascertainment for all Clinical Commissioning Groups in England   Kruskall Wallis p = 0.16

	n	%
White or White British	128,435	88.4
Not stated	5,913	4.1
Asian or Asian British	3,984	2.7
Not known	2,617	1.8
Other	1947	1.3
Black or Black British	1880	1.3
Mixed	548	0.4
Total	*145,324*	

Table A2. Ethnicity profile of the study cohort

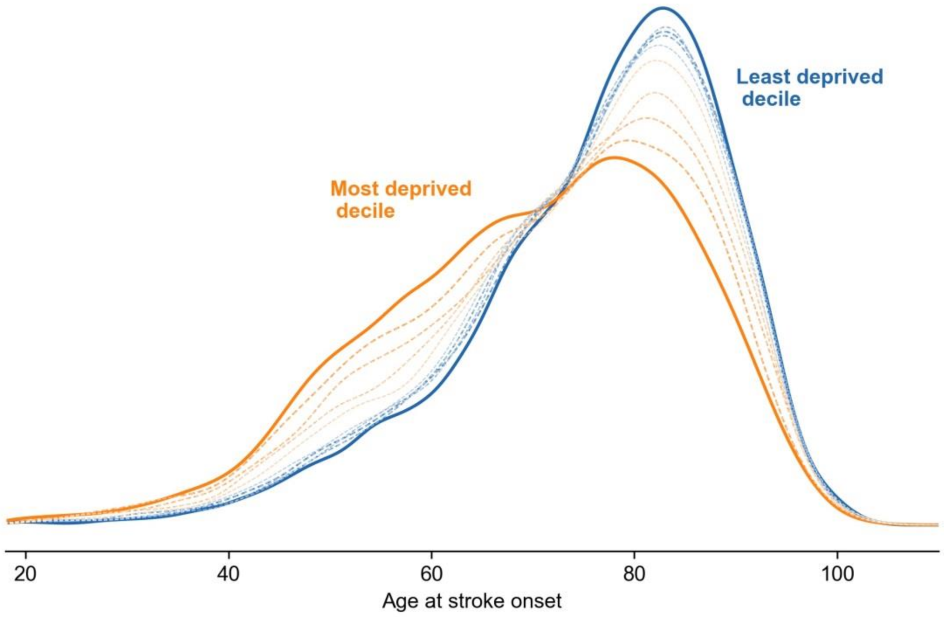

Figure A2. Age distribution (Gaussian kernel density estimate) of patients with stroke , for each decile of Index of Multiple Deprivation

	Least deprived			Decile of Index of Multiple Deprivation						Most deprived
	10	9	8	7	6	5	4	3	2	1
Ischaemic stroke (aIRR, 95% CI)	Ref	1.1 (1.0-1.3)	1.2 (1.0-1.4)	1.2 (1.0-1.4)	1.3 (1.1-1.5)	1.4 (1.2-1.6)	1.5 (1.3-1.7)	1.6 (1.4-1.8)	1.8 (1.5-2.0)	2.0 (1.7-2.3)
Intracerebral haemorrhage (aIRR, 95% CI)	Ref	1.0 (0.9-1.2)	1.0 (0.9-1.2)	1.1 (0.9-1.2)	1.1 (0.9-1.3)	1.2 (1.0-1.4)	1.2 (1.0-1.4)	1.3 (1.1-1.6)	1.5 (1.2 -1.7)	1.6 (1.3 - 1.9)
Ischaemic stroke with pre-stroke diabetes (aIRR, 95% CI)	Ref	1.2 (1.0-1.4)	1.3 (1.1-1.5)	1.4 (1.2-1.7)	1.5 (1.3-1.9)	1.8 (1.5-2.1)	2.0 (1.7-2.4)	2.3 (1.9-2.7)	2.6 (2.2-3.1)	3.2 (2.7-3.8)
Ischaemic stroke with pre-stroke AF (aIRR, 95% CI)	Ref	1.1 (0.9-1.3)	1.2 (0.9-1.4)	1.3 (1.0-1.6)	1.3 (1.0-1.6)	1.2 (1.0-1.5)	1.3 (1.0-1.6)	1.3 (1.1-1.6)	1.4 (1.2-1.8)	1.5 (1.2-1.9)
Ischaemic stroke with pre-stroke hypertension (aIRR, 95% CI)	Ref	1.1 (0.9-1.4)	1.2 (1.0-1.4)	1.3 (1.0-1.5)	1.3 (1.1-1.6)	1.5 (1.2-1.8)	1.6 (1.3-2.0)	1.7 (1.4-2.1)	2.0 (1.6-2.4)	2.3 (1.9-2.8)

Table A3. Age and sex adjusted incidence rate ratios

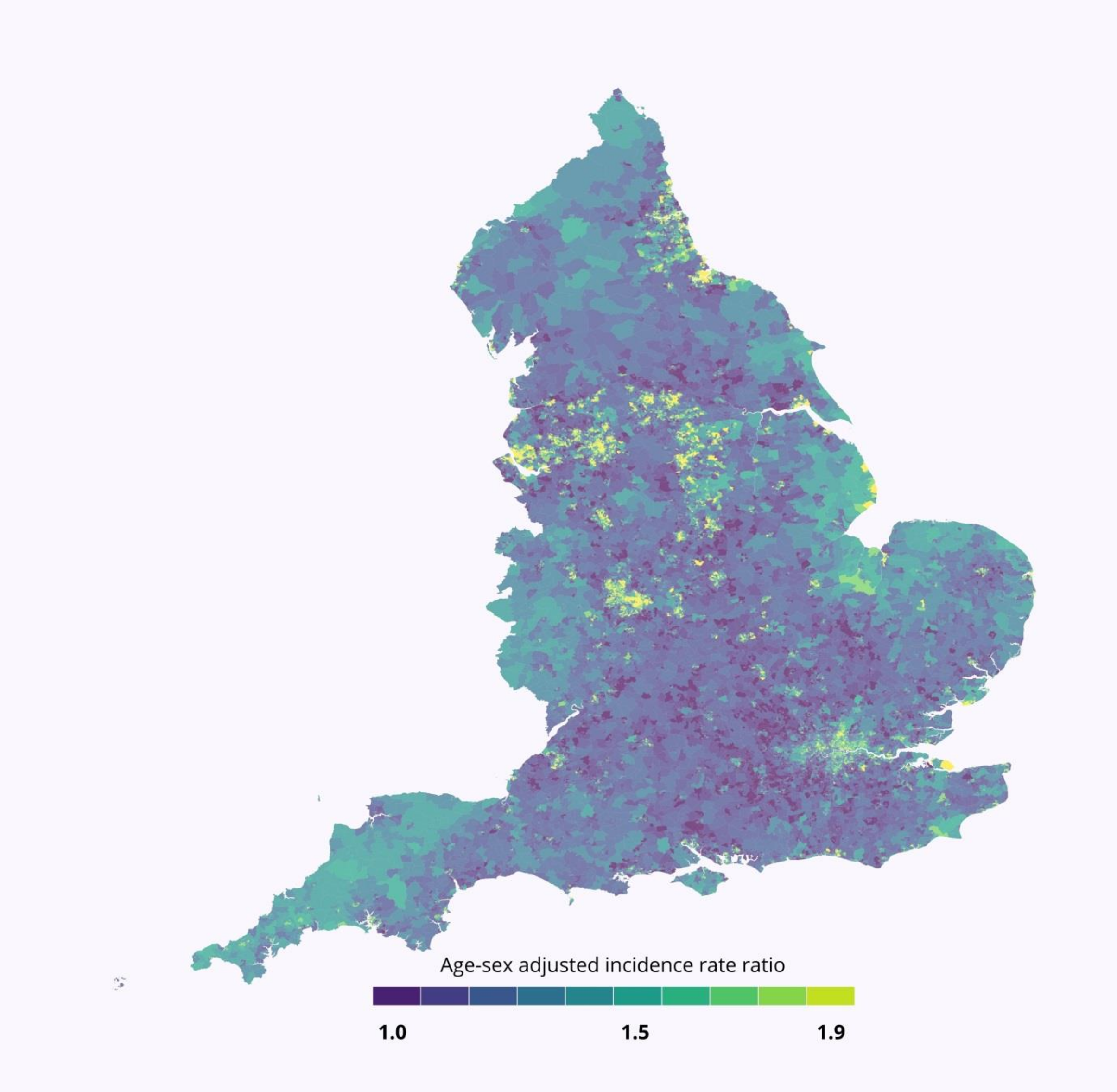

Figure A3. Estimated age and sex adjusted incidence rate ratio of ischaemic stroke for Lower Super Output Area (LSOA) in England. LSOAs have been colour coded according to the estimated age and sex adjusted incidence rate ratio of the corresponding decile of Index of Multiple Deprivation. Image contains data from https://data.gov.uk/dataset/lower_layer_super_output_area_lsoa_boundaries which is licensed under an Open Government license (www.nationalarchives.gov.uk/doc/open-government-licence/version/3/)

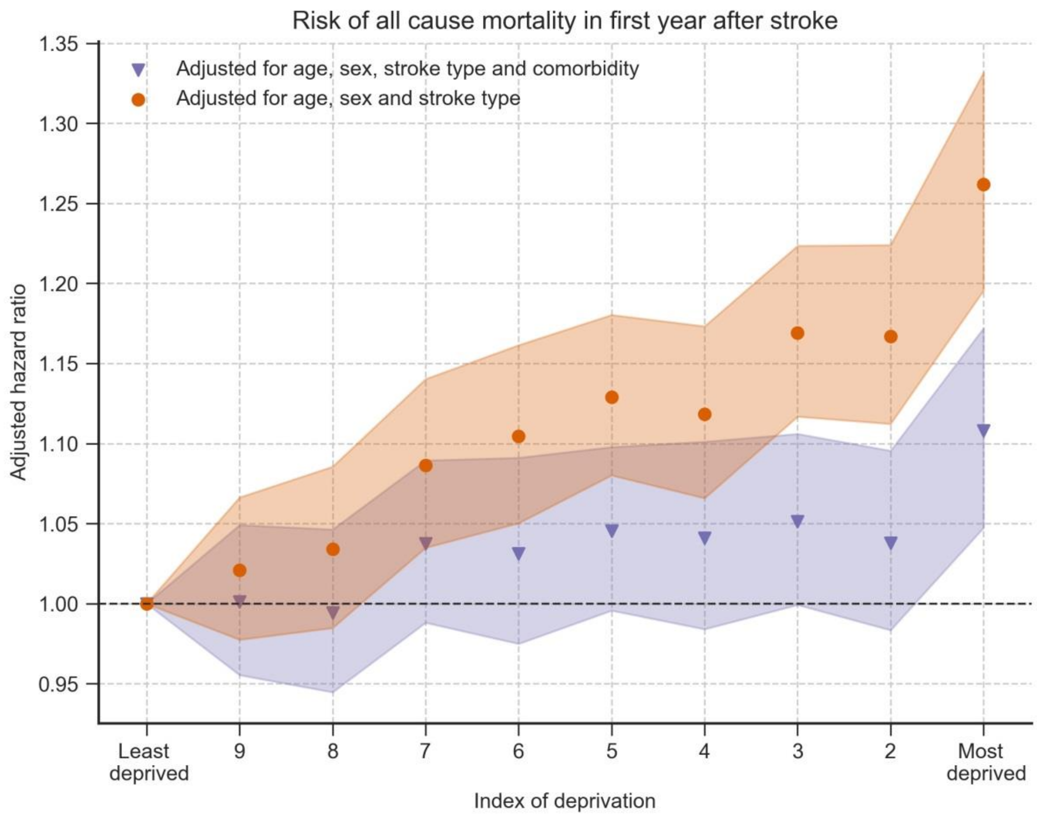

Figure A4. Adjusted hazard ratio of all-cause mortality in the first year after stroke
